# Fractalkine Regulates HEC-1A/JEG-3 Interaction by Influencing the Expression of Implantation-Related Genes in an In Vitro Co-Culture Model

**DOI:** 10.3390/ijms21093175

**Published:** 2020-04-30

**Authors:** Ramóna Pap, Gergely Montskó, Gergely Jánosa, Katalin Sipos, Gábor L. Kovács, Edina Pandur

**Affiliations:** 1 Department of Pharmaceutical Biology, Faculty of Pharmacy, University of Pécs, H-7624 Pécs, Hungary; pap.ramona@pte.hu (R.P.); janosa.gergely@gytk.pte.hu (G.J.); katalin.sipos@aok.pte.hu (K.S.); 2 Szentágothai Research Centre, University of Pécs, H-7624 Pécs, Hungary; montsko.gergely@pte.hu (G.M.); kovacs.l.gabor@pte.hu (G.L.K.); 3 MTA-PTE Human Reproduction Research Group, University of Pécs, H-7624 Pécs, Hungary; 4 Department of Laboratory Medicine, Medical School, University of Pécs, H-7624 Pécs, Hungary

**Keywords:** fractalkine, implantation, endometrium, trophoblast, bilaminar co-culture

## Abstract

Embryo implantation is a complex process regulated by a network of biological molecules. Recently, it has been described that fractalkine (CX3CL1, FKN) might have an important role in the feto–maternal interaction during gestation since the trophoblast cells express fractalkine receptor (CX3CR1) and the endometrium cells secrete fractalkine. CX3CR1 controls three major signalling pathways, PLC-PKC pathway, PI3K/AKT/NFκB pathway and Ras-mitogen-activated protein kinases (MAPK) pathways regulating proliferation, growth, migration and apoptosis. In this study, we focused on the molecular mechanisms of FKN treatment influencing the expression of implantation-related genes in trophoblast cells (JEG-3) both in mono-and in co-culture models. Our results reveal that FKN acted in a concentration and time dependent manner on JEG-3 cells. FKN seemed to operate as a positive regulator of implantation via changing the action of progesterone receptor (PR), activin receptor and bone morphogenetic protein receptor (BMPR). FKN modified also the expression of matrix metalloproteinase 2 and 9 controlling invasion. The presence of HEC-1A endometrial cells in the co-culture contributed to the effect of fractalkine on JEG-3 cells regulating implantation. The results suggest that FKN may contribute to the successful attachment and implantation of embryo.

## 1. Introduction

Embryo implantation, the process of attachment and invasion of the uterus endometrium by the conceptus, is a complex physiological process tightly regulated by multiple biological molecules. Implantation requires a well orchestrated interaction between maternal and foetal tissues and consists of a fine balanced cross talk of cytokines, hormones and chemokines [1,2].

Chemokines play important key roles in several physiologic and pathologic aspects of human reproductive system, among them, the menstrual cycle, ovulation, implantation, cervical ripening, preterm labour and endometriosis [3].

The chemokine fractalkine (FKN) is synthesized as a 373 amino-acid transmembrane molecule. It is the only CX3C-chemokine which has been described [4,5]. FKN exists as both membrane-anchored and soluble forms. As an individual chemokine, its function is not merely chemoattraction, but it also acts as an adhesion molecule and is capable of regulating the immune response via CX3CR1 corresponding receptor interaction [5,6,7].

CX3CR1 belongs to a family of G protein-coupled receptors [7], expressed on cytotoxic effector lymphocytes, including NK cells and cytotoxic T lymphocytes [8]. The FKN-CX3CR1 axis is a crucial regulator of the microglia, the immune cells of the central nervous system (CNS) [9,10].

FKN and its receptor are expressed in numerous reproductive tissues, comprising testis, uterus, ovaries and Fallopian tubes [11,12]. According to previous studies, fractalkine might have an important involvement in the feto–maternal communication during gestation since the trophoblast cells express CX3CR1 and the endometrium cells produce FKN [13,14].

CX3CR1 controls three major signalling pathways, the PLC-PKC pathway, the PI3K/AKT/NFκB pathway and the Ras-mitogen-activated protein kinases (MAPK) pathways (p38, ERK1/2 and JNK) [15]. MAPKs are one of the oldest known signal transduction pathways [16,17] regulating proliferation, growth, migration and apoptosis [18]. PLC-PKC pathway can trigger the MAPK pathway and the secretion of various hormones [15]. The PI3K/AKT signalling pathway is activated via growth factors and plays a critical role in regulating diverse cellular functions including cell growth, metabolism, proliferation, survival, transcription, protein synthesis and mitogenesis [19,20].

Cell migration and invasion are pivotal processes for endometrial cells during implantation, cell trafficking and embryonal morphogenesis [21]. The endometrium undergoes different highly regulated physiological changes during implantation [22,23]. The main supporters of migration are protein kinases, including extracellular signal-regulated protein kinase (ERK)1/2, the phosphatidylinositol 3 kinase (PI3K), the focal adhesion kinase (FAK) and other kinases that can be activated by different cytokines, growth factors and the extracellular matrix [24]. The JNK and p38 pathways are activated in mouse preimplantation development [25].

Progesterone receptor (PR) is involved in the proliferation and differentiation processes by regulating the transcription of specific genes (c-myc, p21, EGFR etc.). The human progesterone receptor has two classical isoforms—A and B—but non-classical intracellular progesterone receptor variants have also been detected (PRC, PRM, PRS and PRT) [26]. PRA and PRB are transcribed from the same gene by using two distinct promoters. The PR can be phosphorylated at basal phosphorylation sites, as well as in response to progesterone. Different protein kinases, including mitogen-activated protein kinases (MAPK), are known to phosphorylate PR at specific serine amino acid residues and modify PR’s activity [27].

Trophoblast invasion into the uterus is an essential step at implantation of the human blastocyst. The action is facilitated by the degradation of the extracellular matrix of the endometrium/decidua by various proteinases, including the matrix metalloproteinases (MMPs) [28]. The tissue inhibitors of matrix metalloproteinases (TIMPs) are key regulators of the metalloproteinases. They inhibit the activity of the MMPs by binding to the highly conserved zinc-binding site of active MMP [29]. It is suggested that MMPs and their regulators control many aspects of reproductive function. The expression of MMP2 and MMP9 depends on the activation of the aforementioned MAPK pathways [30,31] that are regulated by CX3CR1, as well. MMPs have been localized most strongly to the placental bed in early pregnancy suggesting that these proteins are involved in the invasion of trophoblasts [32,33]. MMP expression is also regulated by other factors, e.g., activin signalling and BMP2 signalling pathways [34,35,36].

Activins secreted by endometrial cells belong to the pleiotropic family of the transforming growth factor beta (TGFβ) superfamily of cytokines [37] and are potential factors for maternal–embryo interactions [38]. Activin/Nodal signalling through activin receptors plays an important role at implantation [34,38]. Activin is antagonized by another secreted molecule, called follistatin, which is a key regulator of the biological actions of activin [37]. Follistatins are able to bind directly and irreversibly to activins, neutralizing the ligand [39]. In early development, many processes are contingent on BMPs signalling for cell growth and differentiation [40]. Some follistatin isoforms can bind bone morphogenetic proteins (BMPs) and hinder their biological activities [41].

A bilaminar co-culture system is used to study cell–cell interactions and the technique can be modified for co-culturing any variety of adherent cell types. In this system, the two cell types can connect to each other physically. Therefore, they are able to communicate with each other not only via their released signalling molecules, but also through different cell surface molecules. This way, we can get a better view of action/reaction both of the examined cells [42,43,44,45].

In this study, we focused on the molecular mechanisms governing FKN supporting HEC-1A/JEG-3 interaction and influencing the expression of implantation-related genes in an in vitro co-culture model. Understanding how these mechanisms contribute to implantation might open new targeted medical therapies to give reassurance to women suffering from its failure.

## 2. Results

### 2.1. Effect of Fractalkine on the Viability of JEG-3 Cells in Mono- and Co-Cultures

FKN has been demonstrated to regulate cell proliferation and inhibit apoptosis through different signalling pathways (MAPK, PI3K/AKT) in several cell types (e.g., monocytes, T cells, smooth muscle cells, glia cells, neurons) [10,46,47].

**Figure 1:**
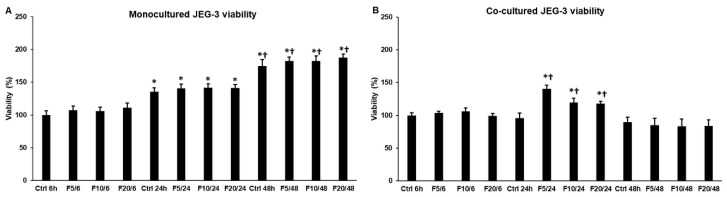
Cell viability determinations of the mono- and co-cultured JEG-3 cells. Viability was measured using CCK-8 cell viability assay after fractalkine treatments. Viability is expressed as percentile of the total cell number of the untreated control cells. (**A**) Viability of the monocultured JEG-3 cells and (**B**) Cell viability measurements of co-cultured JEG-3 cells. Cell viability assays were made in quintuplicate in three independent experiments. The bars represent mean values and error bars represent standard deviation (SD) for three independent experiments (*n* = 3). The * indicate *p* < 0.05 compared to the 6 h untreated control. The † indicate *p* < 0.05 compared to the 24 h untreated control. Abbreviations of fractalkine treatments: 5 ng/mL-F5; 10 ng/mL-F10; 20 ng/mL-F20.

Endometrial HEC-1A cells produce FKN and trophoblast-like JEG-3 cells express fractalkine receptor, CX3CR1; therefore, we supposed that FKN might have an effect on the cell proliferation of JEG-3 cells in both mono- and co-cultures. It was revealed that viability was elevated in the monocultures of JEG-3 cells at 24 h and 48 h, but FKN did not affect the viability compared to the untreated control cells (Figure 1A), suggesting a normal cell proliferation. In case of the co-cultures, FKN influenced cell viability: at 24 h, JEG-3 cells showed significantly increased viability (Figure 1B). Comparing the viabilities of 24 h controls in mono- and co-cultures, it seems that the presence of HEC-1A cells in the co-culture affected JEG-3 proliferation, suggesting that the cell–cell interactions influenced cell cycle and viability (Figure 1A,B). These results indicate that FKN exerts different effects on mono- and co-cultures, and for the co-culture, the effect is time dependent.

### 2.2. Fractalkine Changes the Activation of ERK1/2, p38, JNK and AKT Signalling Pathways in Mono- and Co-Cultured JEG-3 Cells

The increased viability of the cells suggests an enhanced proliferation that is regulated by several signalling pathways. FKN is involved in the regulation of MAPK and PI3K/AKT pathways, regulators of proliferation, differentiation and apoptosis [48,49]. We examined the phosphorylation of ERK1/2, p38, JNK (MAPKs) and AKT to reveal which pathway was affected by FKN and if there were any differences between the activation of signalling pathways with time and by increasing FKN concentrations (5 ng/mL-F5; 10 ng/mL-F10; 20 ng/mL-F20).

**Figure 2:**
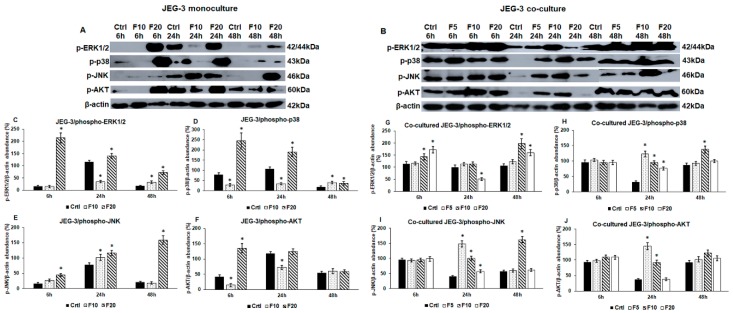
Western blot analyses of signalling pathways regulated by fractalkine in mono- (**A**) and co-cultures (**B**) JEG-3 cells. Cells were collected and pelleted after fractalkine treatments then cells were lysed and their protein contents were measured. The same amount of protein (10 µg) from each sample was separated by SDS-PAGE using 12% polyacrylamide gel, transferred by electroblotting to nitrocellulose membranes. The membranes were probed with anti-phospho-ERK1/2, anti-phospho-p38, anti-phospho-JNK and anti-phospho-AKT according to the manufacturer’s instruction. The experiments were repeated three times. β-actin was used as loading control. (**C**–**F**) Optical density analyses of the examined proteins in JEG-3 monocultures. (**G**–**J**) Optical density analyses of the examined proteins in co-cultured JEG-3 cells. The analyses were carried out using ImageJ software; the optical densities of the examined proteins were expressed as percentage of target protein/β-actin abundance. The bars represent mean values and error bars represent standard deviation (SD) for three independent experiments (*n* = 3). The * mark *p* < 0.05 compared to the appropriate controls (6 h, 24 h and 48 h). Abbreviations of fractalkine treatments: 5 ng/mL-F5; 10 ng/mL-F10; 20 ng/mL-F20.

In case of JEG-3 monoculture, F10 decreased ERK1/2 phosphorylation at 24 h while at 48 h, it increased it compared to the control cells (Figure 2A,C). Meanwhile, F20 treatment increased ERK1/2 phosphorylation at each time points (Figure 2A,C). F10 reduced p-p38 level significantly at 6 h and 24 h but increased again at 48 h (Figure 2A,D), while treatment with F20 caused elevation in p-p38 level at each time points (Figure 2A,D). In contrast with the aforementioned changes of MAPKs, F10 raised p-JNK level at 24 h. Treatment with F20 increased continuously the p-JNK level (Figure 2A,E). AKT showed different alterations compared to MAPKs due to FKN treatment. Phospho-AKT level was elevated by F10 at 24 h while F20 increased it only at 6 h (Figure 2A,F). The results show that the effect of fractalkine is concentration- and time-dependent; the higher FKN concentration (20 ng/mL) has a stronger and longer effect on the protein phosphorylation.

Regarding the co-cultures, in which the two cell types can get in contact with each other, we revealed different alterations in the protein phosphorylation patterns. Although F5 had no effect on the examined signalling pathways in the monoculture, we examined the effect of F5 on the co-cultured JEG-3 cells, too. F5 did not act on the cells at 6 h and 48 h, but it elevated p-p38, p-JNK and p-AKT levels at 24 h that correlated with the increased cell viability (Figure 2B,G–J). F10 raised p-ERK1/2 level at 6 h and 48 h (Figure 2B,G), while the other three pathways were triggered at 24 h and 48 h (Figure 2B,H–J). Interestingly, F20 treatment was less effective on the co-cultured JEG-3 cells. Phospho-ERK1/2 level increased at 6 h and 48 h that was similar to the F10 treatment (Figure 2B,G), although p-ERK1/2 level was reduced at 24 h. Phospho-p38 and p-JNK only elevated at 24 h (Figure 2B,H,I), and p-AKT level did not change during the whole experiment using F20 (Figure 2B,J).

These result reveal that in the case of co-cultured JEG-3 cells, the strongest effect was found at 24 h and at this time point, F5 treatment was the most effective. Comparing the phosphorylation of MAPKs and AKT in mono- and co-cultures it seems that FKN exerts distinct effects on the cells suggesting that the presence of HEC-1A cells in the co-culture modifies the action of FKN.

### 2.3. Fractalkine Exerts Different Effects on the mRNA Expression of Proliferation, Differentiation and Invasion Regulating Genes in Mono- and Co-Cultured JEG-3 Cells

**Figure 3:**
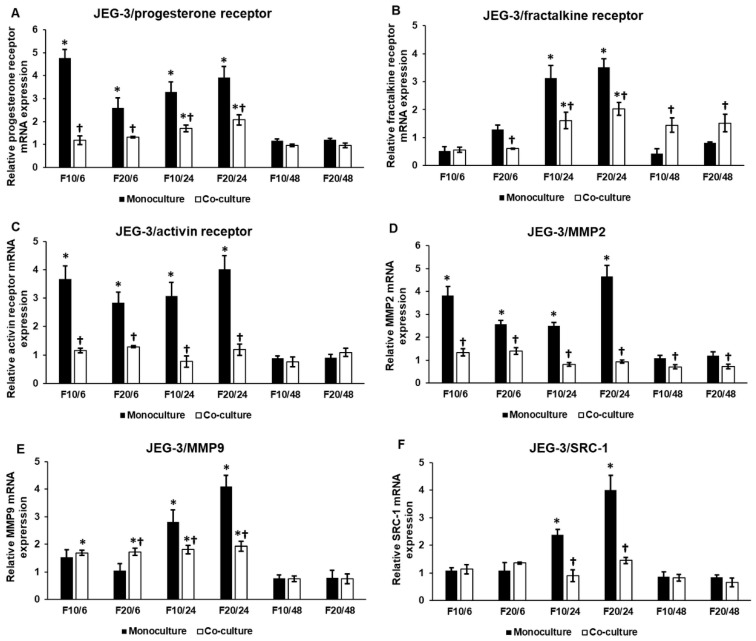
Effects of fractalkine treatments on the mRNA expressions of the implantation-related genes in mono- and co-cultured JEG-3 cells. Real-time PCR was performed with SYBR green protocol using gene specific primers. β-actin was used as house-keeping gene for the normalization. The relative expression of untreated controls was regarded as 1. The mRNA expressions of the treated cells were compared to their appropriate controls (6 h, 24 h or 48 h). (**A**) mRNA expression levels of progesterone receptor. (**B**) mRNA expression levels of fractalkine receptor. (**C**) mRNA expression levels of activin receptor 1B. (**D**) mRNA expression levels of MMP2. (**E**) mRNA expression levels of MMP9. (**F**) mRNA expression levels of SRC-1. The columns represent mean values and error bars represent standard deviation (SD) of three independent determinations (*n* = 3). The * indicates *p* < 0.05 compared to the untreated controls. The † shows *p* < 0.05 between mono- and co-cultures. Abbreviations of fractalkine treatments: 5 ng/mL-F5; 10 ng/mL-F10; 20 ng/mL-F20.

Next, we investigated the mRNA expression levels of PR, which is responsible for differentiation and proliferation of trophoblast cells, and CX3CR1 to reveal if fractalkine provides an autoregulatory effect on its receptor expression on JEG-3 cells. The mRNA expression of PR significantly increased both at 6 h and at 24 h in the monoculture, and was also elevated at the same time points in the co-cultured JEG-3 cells, although there was a significant difference between mono- and co-cultures (Figure 3A). At 48 h, the mRNA expression of PR returned to the control level both in mono- and co-cultured JEG-3 cells (Figure 3A).

CX3CR1 significantly raised at 24 h in the monoculture (Figure 3B). The same phenomenon was found in the co-cultured cells, but in this case, fractalkine maintained the augmented CX3CR1 mRNA expression at 48 h, suggesting a longer effect of FKN on the co-cultured JEG-3 cells through CX3CR1 (Figure 3B).

The activin receptor is supposed to have a role in implantation [50], while matrix metalloproteinases MMP2 and MMP9 are the major contributors to normal implantation by increasing invasiveness [51]. We examined also whether FKN affected the expression of these genes. Interestingly, activin receptor was upregulated in the JEG-3 cells at 6 h and 24 h but its mRNA expression level did not change significantly in the co-cultured JEG-3 cells (Figure 3C), suggesting that the interaction between JEG-3 and HEC-1A cells may influence the effect of fractalkine treatment. The same result was found in case of MMP2 in both mono- and co-cultured JEG-3 cells (Figure 3D). MMP9 mRNA expression was triggered by FKN at 24 h in the monoculture; meanwhile in the co-cultured JEG-3, MMP9 expression began to elevate earlier, at 6 h and remained elevated at 24 h although this level was significantly lower than those in the monoculture (Figure 3E). These results suggest that MMP9 facilitates trophoblast invasion after binding to the endometrial cells while MMP2 expression is downregulated.

We also examined SRC-1 mRNA expression, the co-factor of PR, which is regulated by MAPK pathways and can promote cell differentiation. The expression analysis revealed that SRC-1 mRNA expression only increased at 24 h in the monocultured JEG-3 cells, and there was no significant alteration of SRC-1 mRNA level in the co-culture (Figure 3F).

It seems that in the monocultured JEG-3 cells, F10 exerted a higher effect at 6 h, while at 24 h, F20 was more effective on the gene expression. In case of the co-cultures, the action of FKN did not show concentration dependence.

### 2.4. Western Blot Analysis of the Implantation-Related Genes Reveals Alterations Between Fractalkine Treated Mono- and Co-Cultured JEG-3 Cells

After the mRNA expression analysis, it was unravelled that FKN influenced the expression of genes that are implicated in the implantation process. We also examined if these alterations appeared at protein level, suggesting that FKN regulates implantation by controlling proliferation, differentiation and invasion related proteins of trophoblast cells.

**Figure 4:**
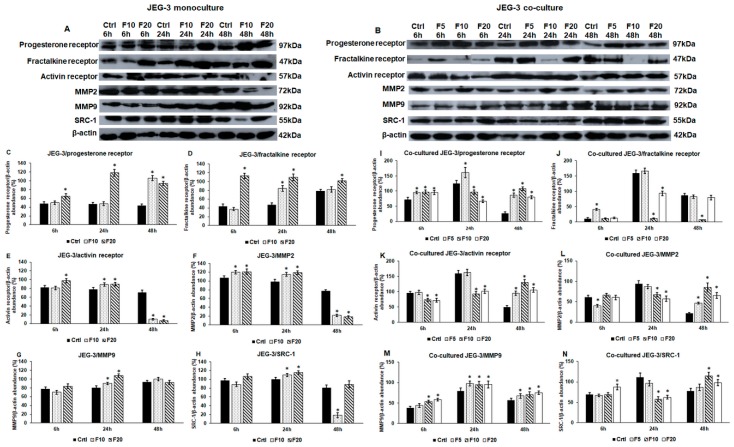
Western blot analyses of implantation-related proteins in fractalkine treated mono- (**A**) and co-cultured (**B**) JEG-3 cells. JEG-3 cells were collected and pelleted after fractalkine treatments. Following lysis, protein contents were measured. The same amount of protein (15 µg) from each sample was separated by SDS-PAGE using 10% or 12% polyacrylamide gel, transferred by electroblotting to nitrocellulose membranes. The membranes were probed with anti-progesterone receptor, anti-fractalkine receptor, anti-activin receptor, anti-MMP2, anti-MMP9 and anti-SRC-1 polyclonal rabbit antibodies according to the manufacturer’s protocols. The experiments were repeated three times. (**C**–**H**) Optical density analyses of the examined proteins in JEG-3 monocultures. (**I**–**N**) Optical density analyses of the examined proteins in co-cultured JEG-3 cells. The analyses were carried out using ImageJ software, the optical densities of the examined proteins were expressed as percentage of target protein/β-actin abundance. The bars represent mean values and error bars represent standard deviation (SD) for three independent experiments (*n* = 3). The * mark *p* < 0.05 compared to the controls. Abbreviations of fractalkine treatments: 5 ng/mL-F5; 10 ng/mL-F10; 20 ng/mL-F20.

In the monocultures, PR and CX3CR1 protein levels showed delays between the elevation of mRNA and protein expressions at F10 treatment, suggesting that the lower FKN concentration has a slower effect on the JEG-3 cells. The effect of F20 lasted longer as the mRNA levels decreased at 48 h but the protein levels were still higher compared to the control cells (Figure 3A,B and Figure 4A).

Activin receptor, MMP2, MMP9 and SRC-1 protein levels correlated with the changes of the mRNA expression levels (Figure 3C–F and Figure 4A), but their expressions varied by time and by FKN concentration. Activin receptor was upregulated by both FKN concentrations at 24 h, and then, at 48 h, the protein level was significantly reduced (Figure 4A,E).

MMP2 protein level raised at 6 h and 24 h and then, at 48 h, the protein level significantly decreased (Figure 4A,F). In case of MMP9, significant elevation was observed at only 24 h (Figure 4A,G). The same alteration was found in case of SRC-1 protein at 24 h, and then F10 decreased SRC-1 level at 48 h (Figure 4A,H). These results show that in the monocultured JEG-3 cells, F20 was more effective in upregulating the protein levels.

In the case of the co-cultured JEG-3 cells, the correlation between mRNA expression and protein level is not as straightforward as for monoculture suggesting that the interaction between the two cell types influences the effect of FKN and the translational rate of the examined proteins.

FKN treatments increased the protein level of PR at 6 h and 48 h, while at 24 h, F5 still increased the expression. On the other hand F10 and F20 had an opposite effect, significantly decreasing the level of PR, showing that the effect of FKN was concentration dependent (Figure 4B,I). SRC-1, a progesterone co-receptor, was elevated by F20 at 6 h, while F10 and F20 treatments reduced SRC-1 protein level at 24 h, and elevated it at 48 h (Figure 4B,N). These changes were parallel to the alterations found at PR. In the case of CX3CR1, the strongest effect was mediated by F10 which significantly reduced CX3CR1 expression at 24 h and 48 h (Figure 4B,J). The activin receptor was downregulated by F10 and F20 at 6 h and 24 h, but it was significantly elevated at 48 h by all three FKN concentrations (Figure 4B,K).

F5 reduced MMP2 protein level at 6 h and increased it at 48 h (Figure 4B,L). F10 and F20 decreased MMP2 level at 24 h and had a reverse effect at 48 h (Figure 4B,L). Interestingly, MMP9 protein level was elevated during the whole experiment by each FKN concentration (Figure 4B,M). This suggests that MMP9 had a specific role in the invasion and may work together with MMP2 at 48 h.

Based on the results, it is proven that F10 and F20 treatments triggered parallel alterations at protein level. The only exception is CX3CR1, which was downregulated by F10 but was upregulated by F20 treatments (Figure 4B,J). The latter result suggests that the regulation of CX3CR1 by FKN is concentration dependent. The fluctuations of PR, CX3CR1 and activin receptor expression may provide protection against the overactivation of signalling pathways regulating the same signal transduction proteins, MAPKs, SMAD transcription factors, the same genes (e.g., MMPs) or cellular process (e.g., invasion). These changes in protein levels can contribute to maintaining the proper implantation mechanism of trophoblast cells.

### 2.5. The Presence of HEC-1A Cells Contributes to the Action of Fractalkine on JEG-3 Cells by Changing the Expressions of Activin, Follistatin and BMP2

**Figure 5:**
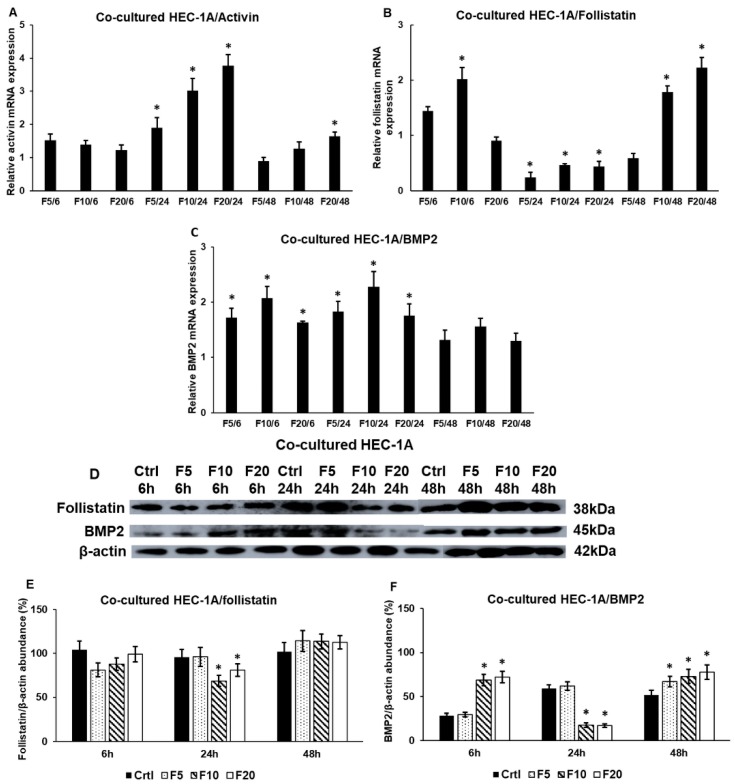
Expression analysis of activin, follistatin and BMP2 in co-cultured HEC-1A cells. Real-time PCR was performed with SYBR green protocol using gene specific primers. β-actin was used as house-keeping gene for the normalization. The relative expression of untreated controls was regarded as 1. The mRNA expressions of the treated cells were compared to their appropriate controls (6 h, 24 h or 48 h). HEC-1A cells were collected by trypsinization directly from the coverslips. The cells were lysed and their protein contents were measured. The same amount of protein (15 µg) from each sample was separated by SDS-PAGE using 10% or 12% polyacrylamide gel, transferred by electroblotting to nitrocellulose membranes. The membranes were incubated with anti-follistatin and anti-BMP2 polyclonal rabbit antibodies according to the manufacturer’s protocols. The experiments were repeated three times. (**A**–**C**) mRNA expression levels of activin, follistatin and BMP2. (**D**) Western blot analyses of follistatin and BMP2. (**E**,**F**) Optical density analyses of the examined proteins in HEC-1A cells. The analyses were carried out using ImageJ software; the optical densities of the examined proteins were expressed as percentage of target protein/β-actin abundance. The columns represent mean values and error bars represent standard deviation (SD) for three independent experiments (*n* = 3). The * mark *p* < 0.05 compared to the controls. Abbreviations of fractalkine treatments: 5 ng/mL-F5; 10 ng/mL-F10; 20 ng/mL-F20.

Based on our results, it seems that the presence of HEC-1A cells in the co-cultures contributed to the action of fractalkine on JEG-3 cells. Therefore, we examined whether HEC-1A-expressed implantation-related genes showed alterations suggesting a cooperation in the implantation process at fractalkine treatment. We determined the mRNA expression levels of activin and follistatin regulating activin receptor, and BMP2 regulating cell growth and differentiation. Interestingly, follistatin and activin levels changed inversely in the co-culture: increased follistatin mRNA level and decreased activin level were found at 6 h and 48 h, and decreased follistatin level and increased activin mRNA level were detected at 24 h (Figure 5A,B). The expression of BMP2 that is also regulated by follistatin binding protein was significantly higher during the whole experiment compared to the control (Figure 5C). These results suggest that the interaction between JEG-3 and HEC-1A cells influences the expressional changes occurring in JEG-3 cells in fractalkine treatment.

At protein level, we found that follistatin level decreased at 24 h that may influence the regulation of activin receptor activity in JEG-3 cells. BMP2 protein level was elevated at 6 h and 48 h. The latter shows that there is a delay between mRNA expression and protein synthesis (Figure 5C,D,F). The decreasing level of BMP2 protein at 24 h is supposed to occur due to the secretion of protein into the culture medium (Figure 5D,F). Our results suggest that HEC-1A cells contribute to the implantation by altering the expression of implantation-related genes and proteins, which could contribute to the action of fractalkine in trophoblast cells.

## 3. Discussion

The early stages of pregnancy comprise the attachment of embryo to the uterine epithelium, invasion of embryo into the uterine stroma and the decidualization of the stroma. Multiple molecules forming a complex network regulate implantation. The interplay between endometrial tissue and conceptus is critical to induce uterine receptivity to implantation [52]. The pre- and early implantation periods require alterations in the gene expression both in the epithelium and in the trophoblast cells. These changes lead to the activation of signalling pathways regulating proliferation, growth, migration and invasion [53].

FKN expression and release were detected in the HEC-1A endometrial cell line [54] while the trophoblast cell line JEG-3 expressed CX3CR1 [14]. Recent publications revealed that FKN might regulate adhesion and migration of trophoblast cells at different stages of pregnancy [14,55] and the FKN-CX3CR1 axis was suggested to be implicated in the maternal-fetal communication [13]. In our study, we established JEG-3 monocultures modelling the pre-implantation period, when the trophoblast cells of conceptus interact with soluble fractalkine secreted by endometrial cells. Bilaminar co-cultures, in which JEG-3 and HEC-1A cells can get in physical contact with each other [10,42,56,57], were used to model the attachment of conceptus with uterine epithelium and the early implantation period. The aim of our study was to unravel the role of fractalkine in the regulation of implantation by the examination of a set of implantation-related genes in trophoblast cells (Figure S1).

Progesterone receptor (PR) is a crucial nuclear receptor regulating implantation and decidualization by the activation of its target genes [58]. The mediators (kinases and coactivators) of PR signalling are also important for successful embryo implantation [53]. During the pre-implantation period, PR initiates a complex signalling network to prepare the endometrium for embryo attachment and implantation that is called the window of receptivity. PR expression in the uterus is dynamically regulated by many factors [58].

We hypothesized that soluble FKN may influence PR expression in trophoblast cells; therefore, the mRNA and protein levels of PR were examined both in mono- and co-cultures. The mRNA expression of PR increased both in the mono- and co-cultures after fractalkine treatments. At protein level, PR showed a significant increase only using the highest FKN concentration at each time point of the JEG-3 monoculture. In the co-culture, PR protein expression revealed fluctuation. These results suggest a time dependent negative feedback in PR expression, and it is supposed that FKN acted in a concentration-dependent manner on PR.

We also examined SRC-1, the member of p160/steroid receptor coactivator family, the coregulatory molecule of PR at FKN treatment [59]. SRC-1 binds to the activated PR in the nucleus and triggers transcriptional response of PR [59]. SRC-1 significantly increased only in monocultured JEG-3 cells at 24 h both at mRNA and protein levels. In case of co-cultured JEG-3 cells, the increment of SRC-1 protein level at 24 h and 48 h showed parallel changes with the PR protein level suggesting the SRC-1 contributed to the PR action.

Activin receptor 1B is expressed by JEG-3 cells showed different expression levels after FKN treatments. Activin receptors are co-localized with activins and follistatins that are the regulators of activin receptor’s activity [60]. Activin receptor regulates SMAD2/3 transcription factors that can be modulated by MAPKs as well [61]. In JEG-3 cells, mRNA expression of activin receptor was significantly elevated after 6 h and 24 h. The protein level of activin receptor followed the changes of the mRNA levels showing that FKN increased the activin receptor expression on the plasma membrane of JEG-3 cells. In the co-culture, the activin receptor B1 protein level showed fluctuation that may be caused by the paracrine effect of activin and follistatin produced by the endometrial cells [60].

MMPs play a critical role in the invasion of trophoblast cells. Among MMPs, MMP2 and 9 seem to be essential in the regulation of invasion and the behaviours of trophoblasts [62,63,64]. During the implantation, trophoblast cells release large amounts of MMP2 and 9 [65]. MMP expression is regulated by many factors e.g., activin signalling, BMP2 signalling and MAPK pathways [33,34,35,36]. FKN treatment increased MMP2 mRNA level at 6 h, MMP2 and MMP9 levels at 24 h only in the monocultured JEG-3 cells. MMP9 was elevated in the co-culture, proposing that MMP9 is more affective in the regulation of invasion than MMP2. At protein level, both MMP2 and MMP9 followed the alterations of mRNA expression in the JEG-3 cells. In the case of the co-cultured JEG-3 cells, we revealed that MMP2 protein level significantly elevated only at 48 h. The increased level of MMP9 protein supports the hypothesis that FKN-CX3CR1 axis acted as a regulator of MMP2 and MMP9 expressions.

These results raise the possibility that during the pre-implantation period (JEG-3 monoculture, at 24 h), fractalkine increases both MMP2 and MMP9 levels to preparing the embryo for implantation. Then at attachment and at early implantation period (co-culture model), MMP9 level increases continuously as the dominant enzyme regulating invasion, while MMP2 participates in the later steps of invasion.

CX3CR1 can be activated both by soluble and membrane-bound FKN. It seems that soluble FKN is important in JEG-3 monocultures acting through CX3CR1 regulating signalling pathways, PLC-PKC, PI3K/AKT/NFκB and MAPK pathways (p38, ERK1/2 and JNK) [15]. These pathways control the proliferation, growth, differentiation and apoptosis [18]. In the case of the co-cultures, both soluble and membrane bound FKN are present, which may contribute to the alterations of fractalkine receptor expression between mono- and co-cultured JEG-3 cells: elevated level in monocultured cells using F10 and 20 at 24 h, but reduced level using the same FKN concentrations at the same time point in co-cultured cells. Fluctuation of the CX3CR1 level in JEG-3 cells may be caused by MAPKs activated by the receptor. MAPKs may activate transcription factors (e.g., SP-1, AP-1, STATs) altering the transcriptional rate of CX3CR1 [66].

To determine the activity of downstream signalling pathways mediated by FKN-CX3CR1, we examined the phosphorylated forms of MAPKs (p38, ERK1/2, JNK) and AKT. The phosphorylation pattern was different in the two examined cell types. In monocultured JEG-3 cells p-ERK1/2 and p-p38 protein levels significantly increased at each time point using F20 although the rate of elevation showed decreasing phenomenon with time. On the contrary, p-JNK showed an increasing level with time, suggesting that p-JNK may replace the aforementioned enzymes when their amounts decrease. F10 exerted lower effect on the JEG-3 cells proposing a concentration dependence. In case of p-AKT, only F20 was able to increase its level. Co-cultured JEG-3 showed increased p-ERK1/2 levels at 6 h and 48 h while p-p38 and p-JNK elevated at 24 h proposing a cooperation between the kinases. In the case of p-AKT, we found similar changes to the p-p38 and p-JNK. The co-work of these three kinases may be responsible for the increased viability of co-cultured JEG-3 cells. Our results prove that FKN provides diverse effects on mono- and co-cultured trophoblasts and these alterations may be caused by the distinct impressions of soluble and membrane-bound FKN and may be influenced by other interactions between trophoblasts and endometrial cells.

It is proven that the presence of HEC-1A in the co-cultures contributes to the action of FKN on JEG-3 cells. This effect is probably mediated by membrane-bound fractalkine expressed by endometrial cells. We examined the expression of those genes and proteins that have been describe as the participants of implantation. We examined activin, follistatin and BMP2 expressions, which are the regulators of activin receptor and BMPR signalling. The activin and follistatin expression changed inversely in HEC-1A cells. The activin mRNA expression increased when the follistatin expression decreased and activin expression decreased when the follistatin mRNA levels were elevated. These results suggest that the interactions between the two cell types may influence the expressions of the activin receptor ligand activin and the binding protein of the ligand follistatin affecting receptor activity. The increasing activin level may contribute to the invasion capacity of trophoblast by acting on matrix metalloproteinase (MMP) expression [34].

Follistatins act as binding proteins for bone morphogenetic protein 2 (BMP2) as well, regulating BMP2 signalling through BMP receptors (BMPR) [67]. The BMP2 signalling inhibits the proliferation of trophoblasts but promotes trophoblast invasion by increasing metalloproteinase secretion [68]. BMP2 is a regulator of SMAD2/3 signalling, influencing the effect of activin receptors, and contributing to increased invasiveness [35,62]. BMP2 induces activin production as well, thus increases the activity of activin receptors [69]. In our experiments, BMP2 mRNA expression was significantly higher during the whole experiment compared to the control. At protein level, BMP2 showed fluctuation suggesting that the changing level of BMP2 in HEC-1A cells contribute to the signalling processes occurring in JEG-3 cells and may increase the action of fractalkine on trophoblast cells. Maybe the increased secretion of BMP2 affects the activity of BMPR elevating MMP expression and invasiveness of trophoblast cells.

Our experiments reveal that increasing fractalkine concentrations act differently on mono- and co-cultured JEG-3 cells. In the case of the monoculture the highest FKN concentration (20 ng/mL) was the most effective in increasing the expression of implantation-related genes. In the case of the co-culture, the intercellular interactions between HEC-1A and JEG-3 cells influenced the action of fractalkine treatment and it seems that both F10 and F20 affected the expression of the examined genes. The activated signalling pathways contributed to cell proliferation and then to the increased invasiveness. The results obtained from our in vitro co-culture experiments should be further investigated in vivo.

**Figure 6:**
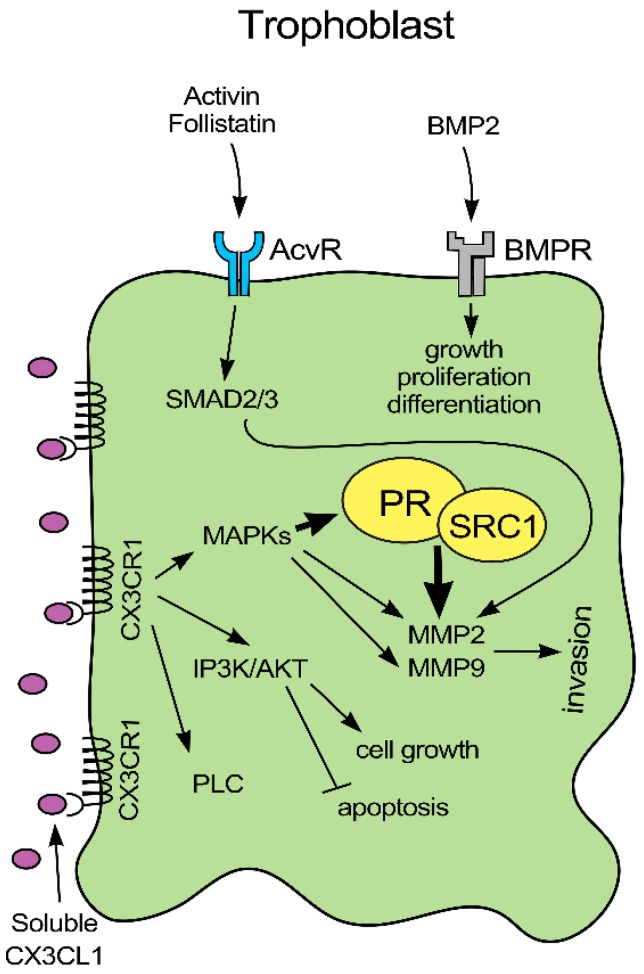
Mechanism of action of fractalkine on JEG-3 trophoblast cells Fractalkine binds to its cognate receptor on the surface of trophoblast cells activating MAPK and AKT pathways. MAPKs can phosphorylate PR on serine amino acid residue increasing its activity. Secreted activin may bind to activin receptor on the surface of trophoblast cells activating SMAD2/3 transcription factors that are also the targets of the BMP2/BMPR signalling pathway. SMAD2/3 transcription factors can upregulate MMP2 and MMP9 expressions. MAPKs are activated by fractalkine signal transduction pathway, act positively on MMP2 and MMP9 transcription. PR influenced by fractalkine can also affect MMP2 synthesis.

In this study, we examined the effect of FKN on the implantation-related genes expressed by trophoblast cells. FKN and CX3CR1 interaction is proven to be involved in the regulation of implantation influencing the expression of several genes (Figure 6). It is revealed that membrane-bound FKN and CX3CR1 receptor binding changes the expression and/or activity of PR by the activation of MAPK pathways [70]. In the co-cultures, it is revealed that HEC-1A cells alter the expressions of activin, follistatin and BMP2 regulators of activin receptor and BMP receptors. Secreted activin may bind to its receptor by paracrine way on trophoblast cells activating SMAD2/3 transcription factors that are also controlled by the BMP2/BMPR signalling pathway. SMAD2/3 pathway can upregulate MMP2 and MMP9 regulating invasion process. MAPKs activated by the FKN signal transduction pathway affect positively MMP2 and MMP9 expression, increasing the invasiveness of trophoblast cells. PR influenced by fractalkine can also modify MMP2 transcription. (Figure 6).

In summary, FKN acts in a concentration and time dependent manner on trophoblast cells. FKN is a positive regulator of implantation, via changing the activity of PR, activin receptor and BMPR and the expression of MMPs. The presence of endometrial cells contributes to the proper implantation as the interactions between trophoblast and endometrial cells in the co-culture changes the gene expression pattern of JEG-3 cells at fractalkine treatment. Based on our results, FKN treatment may contribute to successful attachment and implantation of the embryo.

## 4. Materials and Methods

### 4.1. Cell Cultures and Treatments

The JEG-3 human choriocarcinoma cell line is widely used to study the molecular mechanisms underlying the proliferation and invasive potential of trophoblast cells [54]. The HEC-1A adenocarcinoma derived endometrial cell line is a suitable in vitro model for non-receptive and receptive endometrium [71]. JEG-3 trophoblast-like choriocarcinoma cells (ATCC, HTB-36) were cultured in EMEM medium (Lonza Ltd. Basel, Switzerland) supplemented with 10% fetal bovine serum (FBS, EuroClone S.p.A, Pero, Italy) 1% Non-essential amino acids (NEAA, Lonza Ltd., Basel, Switzerland), 1% sodium-pyruvate (Lonza Ltd., Basel, Switzerland) and 1% Penicillin-Streptomycin (P/S, Lonza Ltd., Basel, Switzerland). HEC-1A endometrial cells (ATCC, HT-112) were maintained in McCoy’s 5A medium (Corning Inc., Corning, NY, USA) supplemented with 10% FBS and 1% P/S. For the experiments, the culture medium was supplemented with charcoal/dextran treated FBS (EuroClone S.p.A, Pero, Italy). For the monocultures, JEG-3 cells were seeded onto culture dishes (60 mm, Corning Inc., Corning, NY, USA) in the appropriate culture medium and were treated after a 24 h resting period. For the co-culture experiments, JEG-3 cells were seeded on culture dishes while HEC-1A cells were placed on Thermanox coverslips (Thermo Fisher Scientific Inc., Waltham, MA, USA). After 24 h, HEC-1A cells were added to JEG-3 cells by turning the endometrial cell holding coverslips upside down facing the trophoblast-like cells [42]. This way the cells were separated only by a thin layer of 1:1 mixture of supplemented EMEM:McCoy’s 5A medium. Both mono- and co-cultures were treated for 6 h, 24 h and 48 h with 5, 10 and 20 ng/mL human recombinant fractalkine (Shenandoah Biotechnology Inc., Warwick, PA, USA). Untreated mono- and co-cultured cells were used as controls.

### 4.2. Cell Viability Assay

The viability of JEG-3 monocultures was measured using Cell-counting Kit-8 (CCK-8) cell viability assay (Merck KGaA, Darmstadt, Germany) after the 6 h, 24 h and 48 h long fractalkine treatments. The viability of the co-culture was determined after the separation of the two cell types. Briefly, the monocultured JEG-3 cells were seeded on a 96-well culture plate. After fractalkine treatments, 10 µL of tetrazolium salt WST-8 reagent was added to each well and the plate was incubated for 1 h at 37 °C and 5% CO_2_. The dehydrogenase reaction was stopped by adding 10 µL 1% sodium-dodecyl sulphate (SDS, Molar Chemicals Kft. Halásztelek, Hungary). The co-culture viability assay was performed in 24-well plate. After each treatment, JEG-3 and HEC-1A cells were separated. JEG-3 cells were incubated for 1 h at 37 °C and 5% CO_2_ in the presence of 40 µL of WST-8 reagent then the reaction was halted by adding 40 µL 1% SDS to each sample. The absorbance of the mono- and co-cultured JEG-3 cells was measured at 450 nm using MultiSkan GO microplate spectrophotometer (Thermo Fisher Scientific Inc., Waltham, MA, USA). Viability was expressed as the percentile of the total cell number of the untreated control cells.

### 4.3. Real-Time PCR

Cells were harvested after washing three times with phosphate buffered saline (PBS, Lonza Ltd., Basel, Switzerland). Total RNA was isolated using Quick RNA mini kit (Zymo Research, Irvine, CA, USA). Complementary DNA was synthesized from 200 ng total RNA using iScript Select cDNA Synthesis Kit (Bio-Rad Inc., Hercules, CA, USA) according to the manufacturer’s protocol. A gene expression analysis was performed with a CFX96 Real-time System (Bio-Rad Inc.) using iTaq™ Universal SYBR^®^ Green Supermix (Bio-Rad Inc., Hercules, CA, USA) in a total reaction volume of 20 µL (7.2 μL of water, 10 μL of 2X Master Mix, 10 μmol/L of forward and reverse primers, and 20 ng of cDNA). Specificity of the primers used in the experiments was determined by generating melting curves after each run. Relative quantification was calculated by the ∆∆Ct (Livak) method using the Bio-Rad CFX Maestro 1.1 software (Bio-Rad Inc., Hercules, CA, USA). β-actin was chosen as reference gene based on the expression analysis of Maestro software, for normalization in each experiment [72]. Relative expression of controls was regarded as 1. Untreated cell controls were made at each examined time point of the treatments, 6 h, 24 h and 48 h, respectively. The mRNA expressions of the treated cells were compared to the appropriate controls. The nucleotide sequences of the primers are described in Table 1.

### 4.4. Immunoblotting

Monocultured JEG-3 cells were harvested after washing three times with PBS (Lonza Ltd., Basel, Switzerland). The co-cultured HEC-1A cells were separated from JEG-3 cells by removing the coverslips from the surface of JEG-3 cells containing culture dishes. The coverslips were washed three times with PBS and the cells were collected by trypsinization. The co-cultured JEG-3 cells were collected from the surface of the culture dish using scraper after washing three times with PBS. Pelleted cells of each sample were lysed with 130 µL of M-PER Mammalian Protein Extraction Reagent (Thermo Fisher Scientific Inc., Waltham, MA, USA) supplemented with Complete mini protease inhibitor cocktail (Roche Ltd., Basel, Switzerland) and PhosSTOP phosphatase inhibitor (Roche Ltd., Basel, Switzerland). The protein contents of the samples were measured with DC Protein Assay Kit (Bio-Rad Inc., Hercules, CA, USA). The same amount of protein (signalling proteins—10 µg, implantation-related proteins—15 µg) from each sample was separated by sodium dodecyl sulphate-polyacrylamide gel electrophoresis (SDS-PAGE) using 10% or 12% polyacrylamide gel, and transferred by electroblotting to nitrocellulose membranes (Pall AG, Basel, Switzerland). The membranes were blocked with 5% (*w*/*v*) non-fat dry milk (Bio-Rad Inc., Hercules, CA, USA) solved in TBST (Tris buffer saline, 0.1% Tween-20) for 1 h at room temperature with gentle shaking. After the blocking step, the membranes were incubated with the following polyclonal rabbit antibodies for 1 h at room temperature in case of FineTest primary antibodies and for overnight at 4 °C in case of the primary antibodies of Cell Signalling Technology: anti-progesterone receptor A/B IgG (1:1000; Wuhan Fine Biotech Co., Ltd., Wuhan, China), anti-fractalkine receptor IgG (1:1000; Wuhan Fine Biotech Co., Ltd., Wuhan, China), anti-activin receptor 1B IgG (1:1000; Wuhan Fine Biotech Co., Ltd., Wuhan, China), anti-matrix-metalloproteinase 2 IgG (1:2000; Wuhan Fine Biotech Co., Ltd., Wuhan, China), anti-matrix-metalloproteinase 9 IgG (1:500; Wuhan Fine Biotech Co., Ltd., Wuhan, China), anti-SRC-1 IgG (1:1000; Wuhan Fine Biotech Co., Ltd., Wuhan, China), anti-Sox17 IgG (1:1000; Wuhan Fine Biotech Co., Ltd., Wuhan, China), anti-BMP2 IgG (1:1000; Wuhan Fine Biotech Co., Ltd., Wuhan, China), anti-phospho-AKT IgG (1:2000; Cell Signaling Technology Europe, Leiden, The Netherlands), anti-phospho-JNK (1:1000; Cell Signaling Technology Europe, Leiden, The Netherlands), anti-phospho-ERK1/2 (1:1000; Cell Signaling Technology Europe, Leiden, The Netherlands) and anti-phospho-p38 (1:1000; Cell Signaling Technology Europe, Leiden, The Netherlands). β-actin (1:2000; Merck KGaA., Darmstadt, Germany) was used as the loading control. Goat anti-rabbit IgG, HRP-linked antibody was used as secondary antibody (1:2000; Cell Signaling Technology Europe, Leiden, The Netherlands) for 1 h at room temperature. The detection of the proteins was carried out with WesternBright ECL chemiluminescent substrate (Advansta Inc., San Jose, CA, USA). Optical densities of Western blots were determined using ImageJ software [73] and was expressed as percentage of target protein/β-actin abundance.

### 4.5. Statistical Analysis

Real-time PCR was carried out in triplicate and cell viability assays were made in quintuplicate in three independent experiments. Western blots are representative of at least three independent experiments. For all data, *n* corresponds to the number of independent experiments. A statistical analysis was performed using SPSS software (IBM Corporation, Armonk, NY, USA). Statistical significance was determined using ANOVA analyzes with Tukey HSD post-hoc tests to compare the treated groups (6 h, 24 h and 48 h) with their appropriate control group (6 h, 24 h and 48 h) and to calculate the significant difference between mono- and co-cultures. The data are shown as mean ± standard deviation (SD). Statistical significance was set at *p* value < 0.05.

## Figures and Tables

**Table 1:** Real-time PCR gene primer list.

Primer	Sequence 5′ → 3′
Progesterone receptor A/B forward	CCAAAGGCCGCAAATTCT
Progesterone receptor A/B reverse	TGAGGTCAGAAAGGTCATCG
Fractalkine receptor forward	CCATTAGTCTGGGCGTCTGG
Fractalkine receptor reverse	GTCACCCAGACACTCGTTGT
Activin receptor 1B forward	CGTTTGCCGTCTTTCTTATC
Activin receptor 1B reverse	ACCAGTTTGATTGGTTCTGT
MMP2 forward	GTCGCCCATCATCAAGTT
MMP2 reverse	GCATCTTCTTTAGTGTGTCCT
MMP9 forward	CGGACCAAGGATACAGTTTG
MMP9 reverse	AAGCGGTACATAGGGTACAT
SRC-1 forward	AGACCCAACCTTTATTCCCA
SRC-1 reverse	GGTGTTACTTGAACAGGCAT
Activin A forward	GAACTTATGGAGCAGACCTC
Activin A reverse	GGACTTTTAGGAAGAGCCAG
Follistatin forward	CAAAGCAAAGTCCTGTGAAG
Follistatin reverse	CCTCTCCCAACCTTGAAATC
BMP2 forward	TAAGTTCTATCCCCACGGAG
BMP2 reverse	AGCATCTTGCATCTGTTCTC
β-actin forward	AGAAAATCTGGCACCACACC
β-actin reverse	GGGGTGTTGAAGGTCTCAAA

## Supplementary Materials

Supplementary materials can be found at https://www.mdpi.com/1422-0067/21/9/3175/s1.

## Abbreviations

AcvR	activin receptor
AKT	protein kinase B
BMP2	bone morphogenetic protein 2
BMPR	bone morphogenetic protein receptor
CNS	central nervous system
CX3CL1	fractalkine
CX3CR1	fractalkine receptor
ERK	extracellular signal-regulated protein kinase
FKN	fractalkine
JNK	c-Jun *N*-terminal kinase
MAPK	mitogen-activated protein kinase
MMP	matrix metalloproteinase
NFκB	nuclear factor kappa-light-chain-enhancer of activated B cells
PI3K	phosphatidylinositol 3 kinase
PKC	protein kinase C
PLC	phospholipase C
PR	progesterone receptor
SMAD2/3	homologues of the Drosophila protein, mothers against decapentaplegic (Mad) and the *caenorhabtidis elegans* protein (Sma)
SRC-1	steroid receptor coactivator-1
