# Supplementary figures and images for: Fractalkine Regulates HEC-1A/JEG-3 Interaction by Influencing the Expression of Implantation-Related Genes in an In Vitro Co-Culture Model
Source: Int J Mol Sci. 2020 Apr 30;21(9):3175. doi: 10.3390/ijms21093175 (PMC7246682; doi:10.3390/ijms21093175)

## Supplementary materials

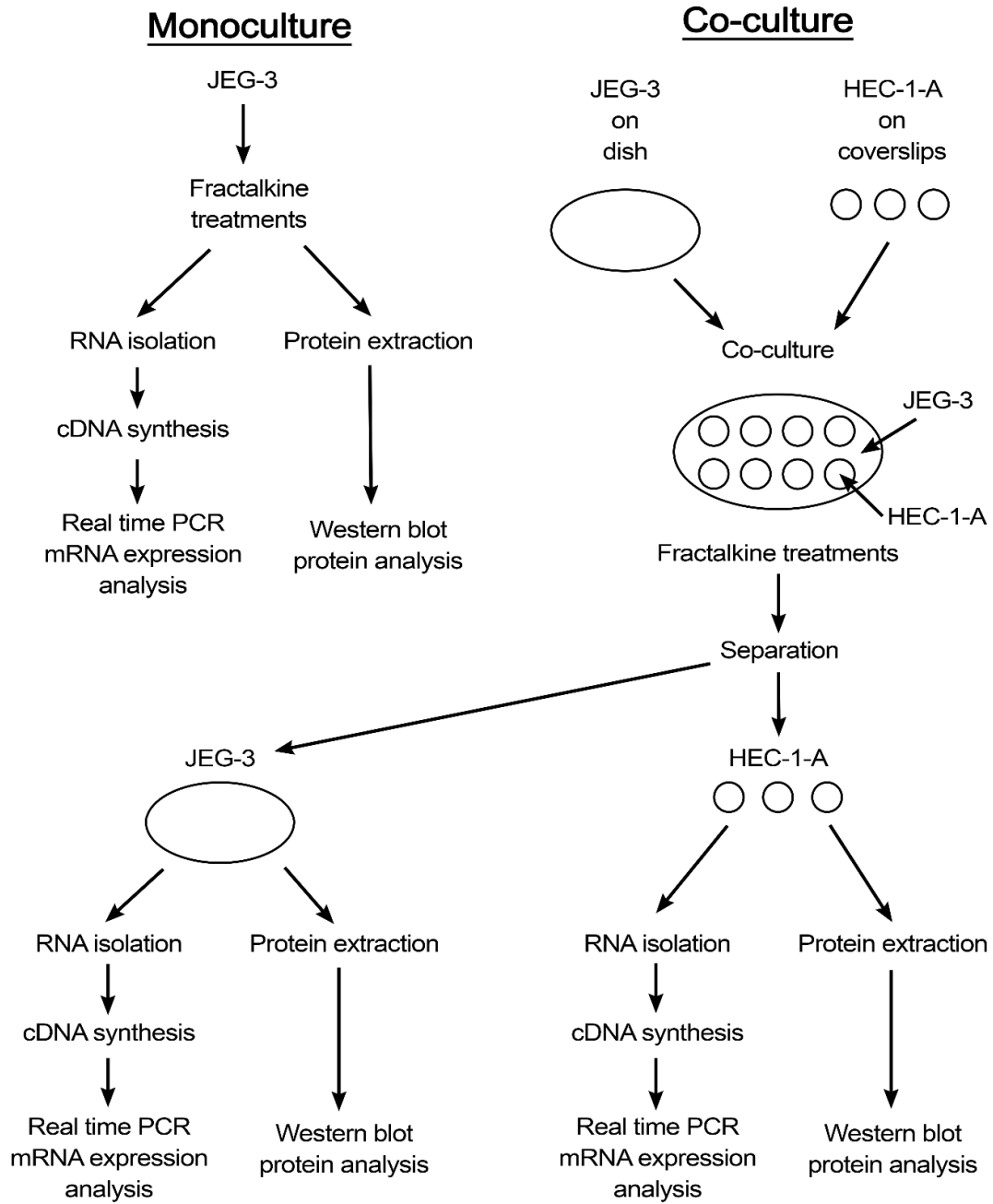

Figure S1. Workflow the experiments.

Supplement: Supplementary file 1 [file ijms-21-03175-s001.pdf]
